# A Novel Carbon Dots/Thermo-Sensitive In Situ Gel for a Composite Ocular Drug Delivery System: Characterization, Ex-Vivo Imaging, and In Vivo Evaluation

**DOI:** 10.3390/ijms22189934

**Published:** 2021-09-14

**Authors:** Lijie Wang, Hao Pan, Donghao Gu, Haowei Sun, Kai Chen, Guoxin Tan, Weisan Pan

**Affiliations:** 1 Department of Pharmaceutics, School of Pharmacy, Shenyang Pharmaceutical University, 103 Wenhua Road, Shenyang 110016, China; wanglijie_a318@163.com (L.W.); gdh15942519001@163.com (D.G.); 13166782785@163.com (H.S.); chenkai19910726@163.com (K.C.); tanguoxintgx@163.com (G.T.); 2 College of Pharmacy, Liaoning University, Shenyang 110036, China; haopan@lnu.edu.cn

**Keywords:** carbon dots, thermosensitive in situ gel, composite ocular drug delivery, pharmacokinetics

## Abstract

We developed a potential composite ocular drug delivery system for the topical administration of diclofenac sodium (DS). The novel carbon dot CD_C-HP_ was synthesized by the pyrolysis of hyaluronic acid and carboxymethyl chitosan through a one-step hydrothermal method and then embedded in a thermosensitive in situ gel of poloxamer 407 and poloxamer 188 through swelling loading. The physicochemical characteristics of these carbon dots were investigated. The results of the in vitro release test showed that this composite ocular drug delivery system (DS-CD_C-HP_-Gel) exhibited sustained release for 12 h. The study of the ex vivo fluorescence distribution in ocular tissues showed that it could be used for bioimaging and tracing in ocular tissues and prolong precorneal retention. Elimination profiles in tears corresponded to the study of ex vivo fluorescence imaging. The area under the curve of DS in the aqueous humor in the DS-CD_C-HP_-Gel group was 3.45-fold that in the DS eye drops group, indicating a longer precorneal retention time. DS-CD_C-HP_ with a positive charge and combined with a thermosensitive in situ gel might strengthen adherence to the corneal surface and prolong the ocular surface retention time to improve the bioavailability. This composite ocular delivery system possesses potential applications in ocular imaging and drug delivery.

## 1. Introduction

Due to the growth and ageing of the population as well as the rapid development of automated technology, the number of people suffering from optical diseases has increased significantly and is showing a younger trend [1]. Eye diseases seriously affect human health and quality of life [2]. Ophthalmic inflammation is one of the most common diseases in our daily lives, but it is also one of the most overlooked. The first line of treatment for ophthalmic inflammation is broad-spectrum antibiotics [3]. However, prolonged use of antibiotics will lead to bacterial resistance, and antibiotics abuse can trigger drug-derived eye diseases and further cause eye damage. Thus, diclofenac sodium (DS), a nonsteroidal anti-inflammatory drug (NSAID), is the most widely used in some countries as a model drug [4,5]. DS eye drop solutions are the main dosage form for relieving ocular inflammation. However, its bioavailability is only 5% due to the multiple protective barriers in the eye. Therefore, there is an urgent need for a formulation design that can reduce the frequency of dosing and improve the bioavailability of DS through continuous ocular delivery and improved ocular penetration.

Due to the special physiologic and anatomical structure of the eye, most conventional ophthalmic drugs have a short residence time in front of the cornea, and frequent dosing is required, leading to low bioavailability and poor patient compliance [6]. Therefore, ocular drug delivery should have enough adhesion to prevent surface tear washing, and should have appropriate lipophilicity and hydrophilicity to be able to penetrate the tear barrier into the cornea [7]. However, when the drug reaches the highly organized cornea, it has an anatomical barrier mainly composed of three primary layers, i.e., epithelium (adjacent to the conjunctiva), stroma (the middle layer), and endothelium (the innermost layer) [6,8]. All layers of a corneal barrier act as a certain selective barrier for lipophilic and hydrophilic drugs. In addition, intercellular tight junctions on the epithelium limit the diffusion of hydrophilic substances (size > 100 Da) through the paracellular route [9,10,11]. The ophthalmic in situ gel system is comprised polymers that alter their conversion from sol to gel according to the environmental stimuli of the eye like temperature, pH, and ionic strength [8]. Due to that nanoparticle-based thermosensitive in situ gel composite systems have simple preparation and drug loading processes, are low cost, and are easy to scale-up, they have been the most extensively used composite drug delivery systems [7].

Due to unique properties [8] of nanoparticle systems, several types of nanoparticles [12,13,14] have been embedded in hydrogels to design sustained release composite systems for ophthalmic drug delivery. Carbon dots (C-dots) are spherical nanoparticles with particle sizes less than 10 nm that have excellent water solubility, good fluorescence, ease of synthesis and modification, small size, low toxicity, and good biocompatibility [15,16,17]. These characteristics of C-dots have prompted them to be applied in the medical fields of biosensing, detection of drugs and other molecules, drug delivery and biological imaging [18]. However, the application of carbon dots in the eyes is rarely reported. Jian et al. reported CQD_S_ prepared by spermidine as a potential antibacterial candidate for the treatment of infectious keratitis [19]. The one-step hydrothermal method is a green synthetic strategy with environmentally friendly solvents using simple equipment and has become the main method for the preparation of carbon dots in large quantities [17]. Even though many substances are used as carbon sources [20] to prepare C-dots, materials with a high yield mass, environmental friendliness, self-targeting, and good biocompatibility are still essential for C-dots to be applied in the medical field.

Hyaluronic acid (HA), also known as Restylane, is a linear macromolecule acid mucopolysaccharide widely distributed in humans and animals [21]. In the human body, it exists in the skin and connective tissue as an extracellular matrix. In addition to providing water and volume for cells, it also has the characteristics of tissue stability, a strong binding force, high viscoelasticity, weak species and tissue diversity, and no immunogenicity [22]. With good water solubility and biocompatibility, HA is widely used in the fields of drug delivery, ophthalmology, arthritis, cancer treatment, and health food [23]. In addition, HA has a strong affinity for specific substances on the cell surface, such as the CD44 receptor [24]. The CD44 receptor is overexpressed on the surface of various tumor cells and eye cells under inflammatory conditions [25], and thus, HA is often used as a target material to deliver drugs. It has been reported that as a carbon source [20,24,26,27], HA was added with a passivator to prepare CDs as self-targeting CD_HA_ and successfully delivered the loaded drug into tumor cells and was bioimaged. Carboxymethyl chitosan (CMC) is a water-soluble chitosan derivative with many characteristics, such as strong antibacterial properties, a fresh-keeping effect, and acting as an amphoteric polyelectrolyte [28]. There are many applications in cosmetics, preservation, medicine, etc., [29,30]. Numerous reports have described chitosan as a carbon source to prepare carbon dots. However, the poor water solubility of chitosan limits its application as a carbon source to prepare C-dots [31,32,33]. CMC, because of the introduction of carboxymethyl groups, destroyed the secondary structure of chitosan molecules and greatly reduced its crystallinity so that it became almost amorphous and had better water solubility [30]. Taken together, this article chooses CMC as the carbon source and HA to jointly synthesize high-yield mass and environmentally friendly C-dots.

**Scheme 1:**
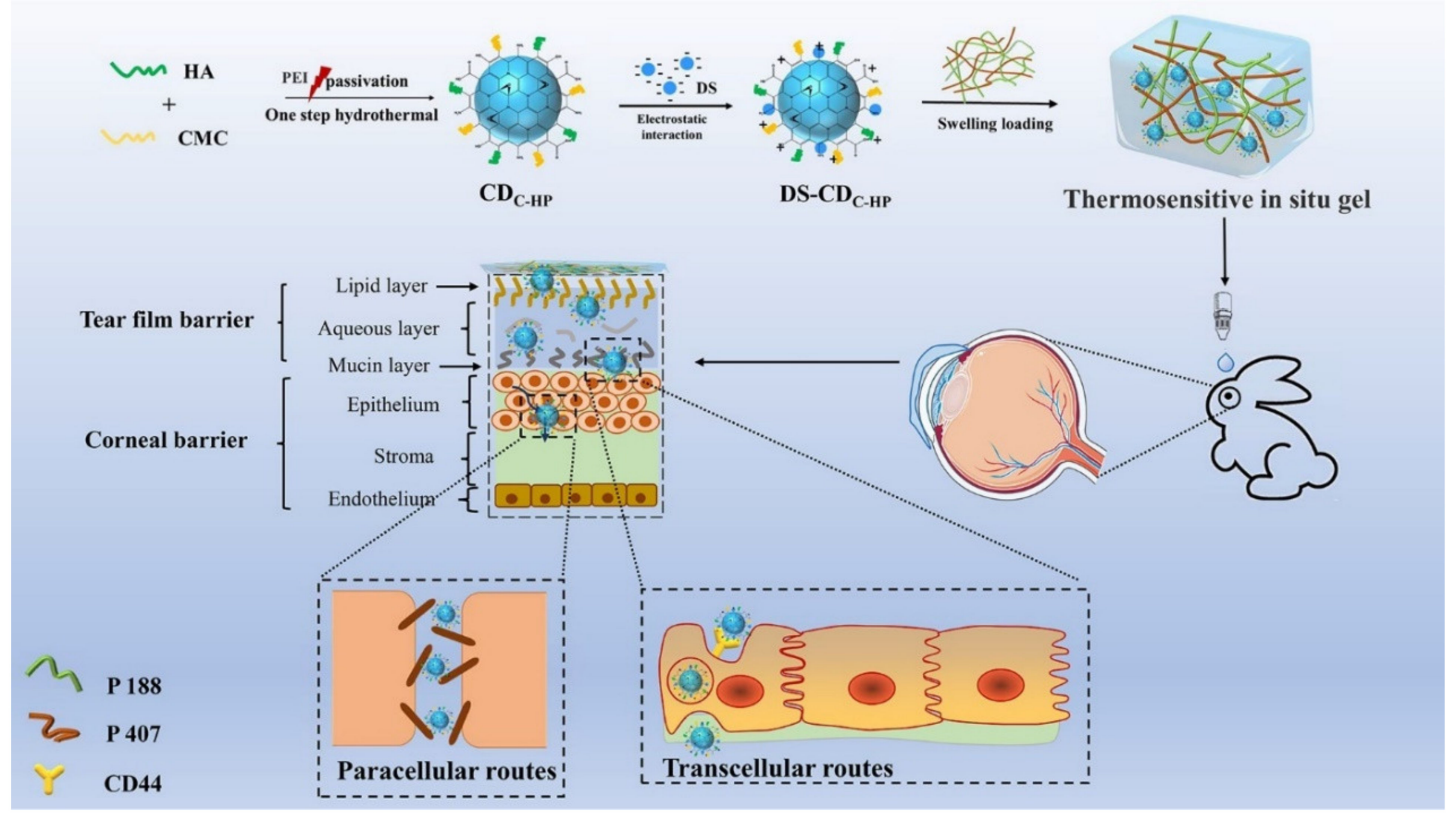
Illustration of fabricating and the possible process in vivo and penetration behavior of DS-CD_C-HP_-Gel. The penetration behavior might be induced by transcellular routs and paracellular routs.

In this work, C-dots and temperature-sensitive hydrogels were designed to prepare a composite ocular drug delivery system. It is expected that the combination of C-dots with positively charged and thermosensitive gels can improve ocular surface retention and penetration to improve the bioavailability of DS. To prepare this drug delivery system, novel C-dots (CD_C-HP_) were synthesized (shown in Scheme 1) by a simple one-step hydrothermal method of natural biomolecular materials (HA and CMC) with a passivation agent of polyethyleneimine (PEI) which was used to enhance the fluorescence intensity of CD_C-HP_. CD_C-HP_ and DS which was selected as a model drug, combined through electrostatic interactions to form DS-CD_C-HP_. To further improve the DS duration and bioavailability from the DS-CD_C-HP_ nanoparticle system, DS-CD_C-HP_ was embedded in thermosensitive hydrogels of poloxamer to form a composite ocular drug delivery system. The in vitro and in vivo results show great potential for ophthalmic imaging and drug delivery.

## 2. Results

### 2.1. Preparation and Characterization of CD_C-HP_

**Figure 1:**
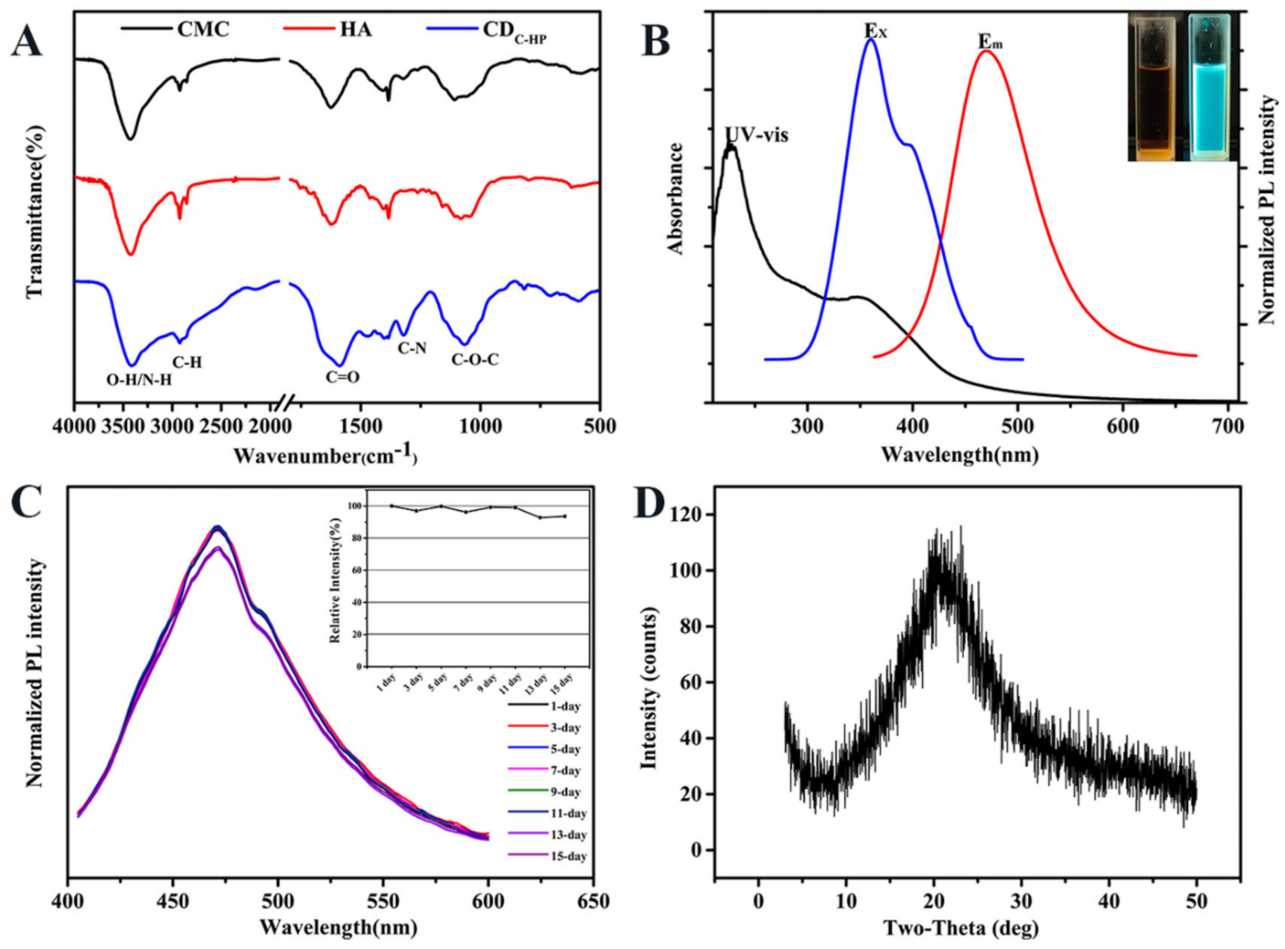
FT-IR spectrum of CMC, HA, and CD_C-HP_ (**A**); UV-vis absorption, excitation and emission spectra of CD_C-HP_ (**B**); The inset in Panel B: optical images obtained under daylight (**left**) and a UV beam of 365 nm (**right**); The photo stability of CD_C-HP_ at different time (**C**); XRD pattern of CD_C-HP_ (**D**).

Quinine sulfate was selected as a reference sample to calculate the fluorescence quantum yield [27]. The fluorescence quantum yield and mass yield of CD_C-HP_ were 16.20% and 55.43%, respectively. The preparation of CD_C-HP_ is shown in Scheme 1. FT-IR spectra were chosen to determine the functional groups on CD_C-HP_. As exhibited in Figure 1A, the broad characteristic peaks from 3000 to 3690 cm^−1^ were attributed to the stretching vibrations of the N-H and O-H groups. The peaks observed at 2923 cm^−1^ correspond to C-H stretching vibrations. The typical absorption peaks centered at approximately 1632 cm^−1^, 1402 cm^−1^, and 1066 cm^−1^ were attributed to the vibrations of C=O, C-N and C-O-C, demonstrating the existence of amido and ester bonds.

The optical properties of CD_C-HP_ were evaluated via ultraviolet-visible (UV-Vis) and photoluminescence (PL) measurements. As shown in Figure 1B (inset), the yellow–orange aqueous solution of CD_C-HP_ revealed strong blue emission under 365 nm UV irradiation. The UV-vis spectrum of an aqueous solution of CD_C-HP_ showed a sharp peak at a wavelength of 225 nm and broad absorption ranging from 200 nm to 500 nm, which were assigned to the π − π * transition of sp^2^ hybridization and the n − π * transition of the C=O group of CD_C-HP_, respectively. The maximum excitation and emission wavelengths of the aqueous solution of CD_C-HP_ were fixed at 357 and 473 nm (Figure 1B), respectively. These optical properties of CD_C-HP_ resulted from surface groups on the CD_C-HP_, which agreed with the FT-IR results. Figure 1C reveals that the emission behavior of CD_C-HP_ in this period of time is almost the same in PL intensity, which suggests that photobleaching of CD_C-HP_ would not occur.

**Figure 2:**
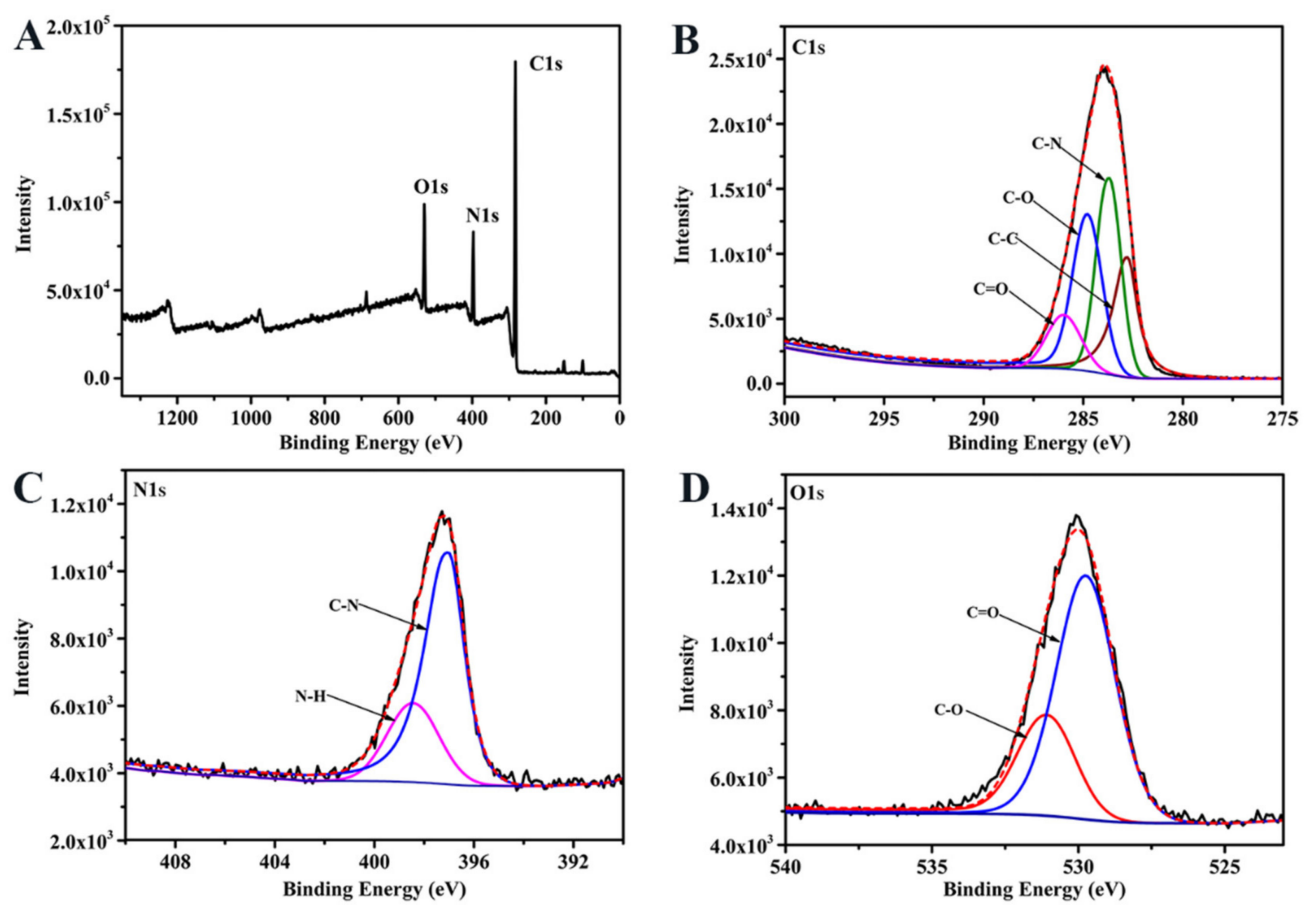
XPS scanning spectrum of CD_C-HP_ (**A**) and high resolution spectrum of C 1s (**B**), N 1s (**C**), and O 1s (**D**).

The elemental composition and structure of CD_C-HP_ were determined by XPS measurements. Figure 2A shows that the peaks at 284.6 eV, 398.5 eV, and 531.3 eV in the XPS spectrum were attributed to C 1s, N 1s, and O 1s, respectively. As shown in Figure 2B, the C 1s spectrum showed four peaks at 282.5 eV, 284.5 eV, 285.4 eV, and 286.5 eV, which correspond to the C-C, C-N, C-O, and C=O groups of CD_C-HP_, respectively. The N 1s spectrum of CD_C-HP_ (Figure 1C) showed two distinct peaks at 399.8 eV and 401.3 eV, which correspond to C-N and N-H groups. The O 1s spectrum exhibited two peaks at 530.2 eV and 531.5 eV, which correspond to C=O and C-O groups. These FT-IR and XPS results showed -C=O, O-H and C-O-C groups in CD_C-HP_, which were specific functional groups of HA (Figure 1A).

**Figure 3:**
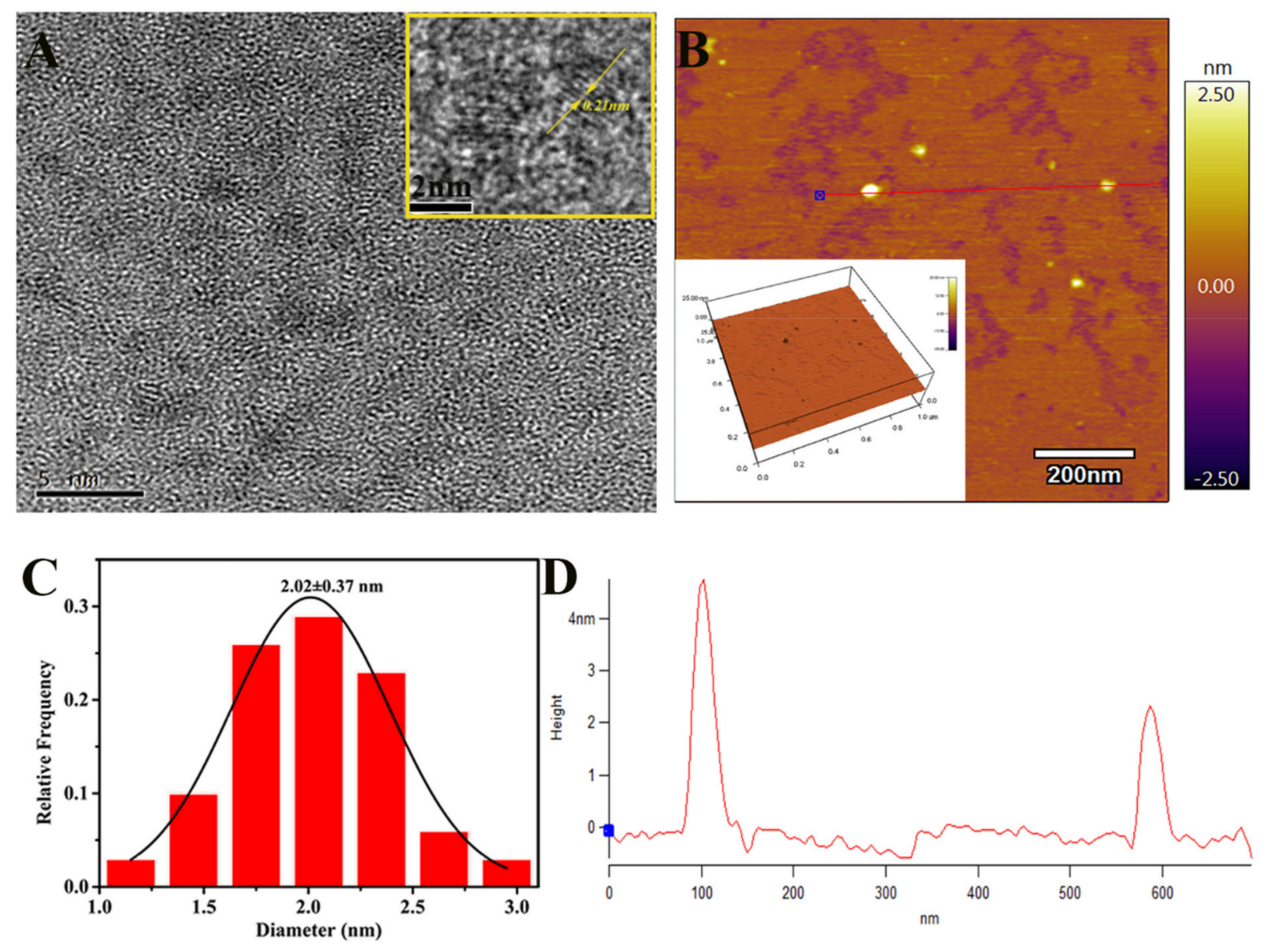
TEM image of CD_C-HP_ (**A**), inset: HRTEM image of a single CD_C-HP_ particle; AFM topography image of CD_C-HP_ (**B**), inset: AFM 3D image; particle size distribution histogram of TEM image of CD_C-HP_ (**C**); AFM topography image; Height profile along the line in AFM topography image (**D**).

The precise size and morphology of CD_C-HP_ in full detail was characterized by TEM and AFM imaging techniques. Figure 3A shows that CD_C-HP_ is spherical with an average statistical diameter of 2.02 ± 0.37 nm (Figure 3C) and has good dispersibility. The high-resolution TEM image (Figure 3A, inset) exhibited a crystalline structure with a lattice distance value of 0.21 nm, which corresponds to the (100) diffraction facets of graphitic carbon [34], suggesting that CD_C-HP_ has a graphite-like structure [35]. The AFM images (Figure 3B,D) indicated that the CD_C-HP_ presented a spherical shape, and the average height was 2–4 nm, which was similar to the TEM characterization. The XRD pattern (Figure 1D) suggested that CD_C-HP_ has a broad diffraction peak at 2θ = 21.5°, suggesting that CD_C-HP_ was an amorphous structure [36]. After the structural and morphological characterization of CD_C-HP_, we further evaluated their charge via measurement of the zeta potential (ζ). As shown in Table 1, the zeta potential of CD_C-HP_ was +38.33 mV, suggesting an electrostatic attraction between CD_C-HP_ and diclofenac sodium (DS, −17.17 mV).

As an ocular drug delivery system, carbon dots made from natural biomaterials have surface modification and antibacterial activity that can retard the undesired effects, enhance biocompatibility and the functionality of nanoparticles. Based on the bacteriostatic ability of carbon dots reported in previous, the bacteriostatic ability of novel CD_C-HP_ carbon dots was also investigated in this study. Three kinds of bacteria commonly found in eye infections were selected, such as *E. coli*, *S. aureus,* and *P. aeruginosa*, to investigate the bacteriostatic ability of carbon dots. The ability of CD_C-HP_ to inhibit bacterial growth was tested by a disk diffusion assay, and the MIC value of CD_C-HP_ was also investigated. Figure S1 shows that CD_C-HP_ has a certain antibacterial effect (approximately 0.3 mg/mL CD_C-HP_ against *E. coli*, *S. aureus,* and *P. aeruginosa*). The results showed that CD_C-HP_ carbon dots have antibacterial ability, which is of great significance for the development and the storage of carbon dots for ocular drug delivery applications. In addition, the cell imaging of CD_C-HP_ in corneal epithelial cells (HCE-T) was observed by inverted fluorescence (IX7I, Japan) and showed that it was mainly distributed in the cell membrane and cytoplasm with green fluorescence and has CD44 targeting characteristics (Figure S2). The in vitro cytotoxicity showed that CD_C-HP_ had no cytotoxicity (Figure S3) in the model cells (MCF-7, NIH-3T3, HCE-T and HRPEC cells).

### 2.2. Physicochemical Characterization of Formulations

#### 2.2.1. Characterization of Nanoparticles

**Figure 4:**
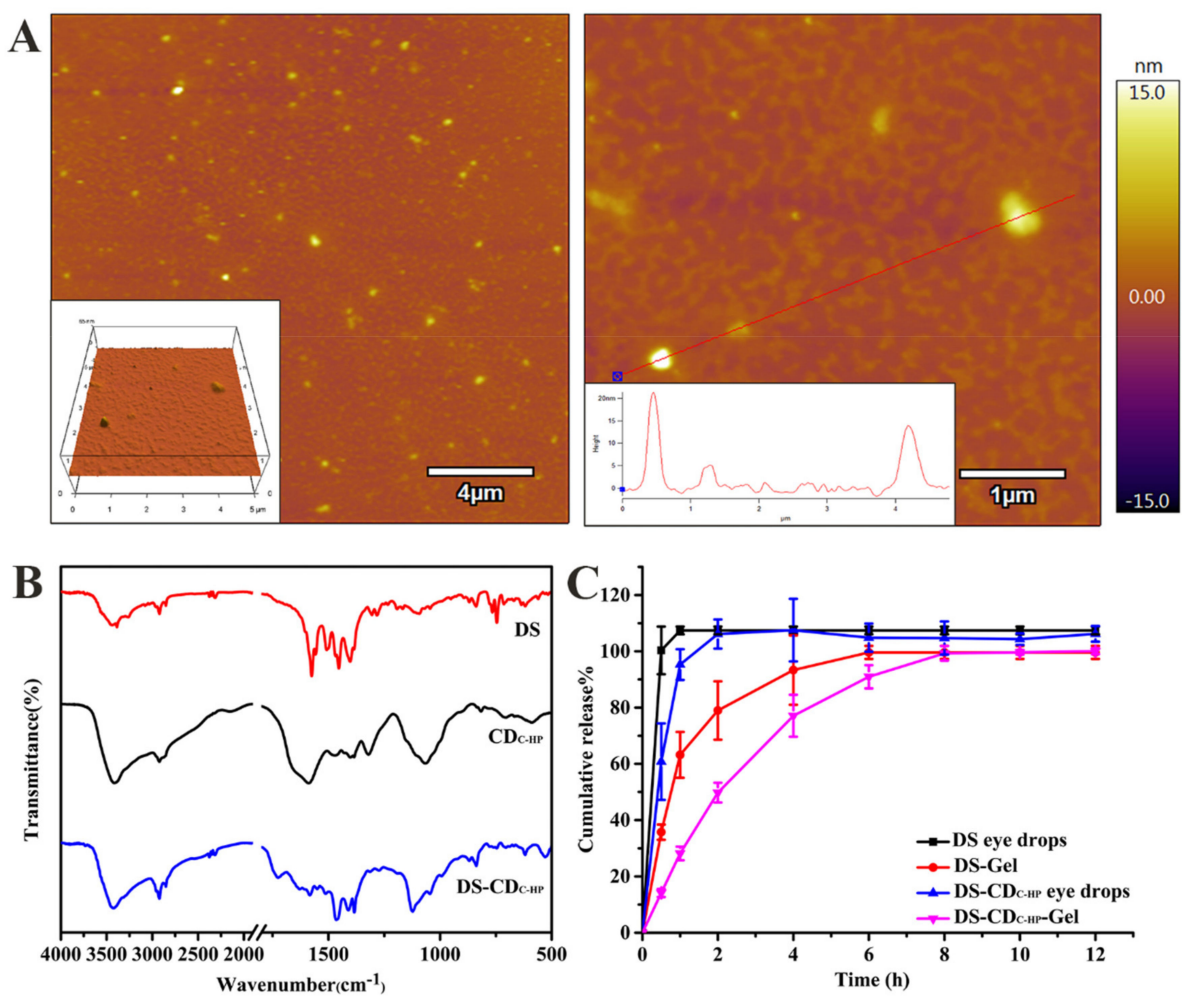
AFM images of DS-CD_C-HP_ (**A**); FT-IR spectrum of DS, CD_C-HP_ and DS-CD_C-HP_ (**B**); in vitro release profiles of DS eye drops, DS-Gel, DS-CD_C-HP_ eye drops and DS-CD_C-HP_-Gel (**C**).

The zeta potential (ζ), particle size (PS), particle size distribution index (PDI), drug loading efficiency (DLE), and drug loading content (DLC) of DS-CD_C-HP_ are displayed in Table 1. It is generally recognized that as an ocular drug delivery system, particles with small sizes are more comfortable to the ocular tissue and reduce the risk of irritation. DS-CD_C-HP_ exhibited a size of 90.12 ± 2.16 nm in Table 1, which was smaller than 200 nm (less than 200 nm can be easily filtered for sterilization), increased the stability during storage and was proper for ocular application. The PDI value of DS-CD_C-HP_ was 0.11 ± 0.02 (lower than 0.3), suggesting good dispersion quality. The shape of the CD_C-HP_ nanoparticles was characterized by AFM. Figure 4A shows that the majority of the particles were spherical in shape. FT-IR spectra of DS, CD_C-HP_ and DS-CD_C-HP_ are shown in Figure 4B. In the spectrum of DS, the characteristic peaks observed at 1575 cm^−1^, 1507 cm^−1^ and 1453 cm^−1^ were attributed to the stretching vibrations of the carboxyl ions of C=O, C=C and COO groups. The typical absorption peaks centered at 766 cm^−1^ were attributed to the stretching vibrations of C-Cl. In the FT-IR spectrum of DS-CD_C-HP_, all of the main characteristic bands of CD_C-HP_ were found in the spectrum. In contrast, the characteristic peak of DS at 1575 cm^−1^ disappeared, demonstrating the existence of hydrogen bonding between DS and CD_C-HP_. DS-CD_C-HP_ was kept in the dark at 4 °C, and the change in the particle size distribution (Figure S4) was measured at different times by a Malvern Zetasizer (UK). The results showed that DS-CD_C-HP_ had good storage stability.

#### 2.2.2. Drug Loading and Release

The DS loading efficiency (DLE) and drug loading content (DLC) of DS-CD_C-HP_ were 84.37 ± 0.33% and 30.68 ± 0.10% (Table 1), respectively. This outcome indicated that there was a strong electrostatic interaction between DS and CD_C-HP_, which is consistent with the zeta potential results. The gelling temperature of DS-CD_C-HP_-Gel was 34.3 °C, and the viscosity was 14.33 mPa·s, indicating that this composite drug delivery could form a gel on the ocular surface after administration, increase adhesion and sustain drug release.

The in vitro release profiles of DS from these formulations are illustrated in Figure 4C. The release pattern of DS-CD_C-HP_-Gel was a biphasic release: initially a burst release appeared followed by a sustained release. This result might be due to the difference in the rate of desorption and diffusion in the gel layer between the free DS on the surface of CD_C-HP_ and the strongly electrostatically bound DS on CD_C-HP_. Biphasic drug release behavior is very beneficial in ocular drug delivery, as it rapidly facilitates the therapeutic concentration of the drug initially and afterwards maintains a sustained release for a prolonged period. In addition, as shown in Figure 4C, the release rate of DS from the DS-CD_C-HP_ eye drops was slower than that of DS eye drops. This might be attributed to the strong electrostatic interaction between the drug and carrier in the DS-CD_C-HP_ formulation. This consequence also indirectly proves that the release rate of DS from DS-CD_C-HP_-Gel is obviously slower than that from DS-Gel. DS released from DS-CD_C-HP_-Gel was 50% after 2 h and 90% after 6 h. In comparison to the other three release profiles of DS eye drops, DS-Gel and DS-CD_C-HP_ eye drops, the developed DS-CD_C-HP_-Gel is a proper platform for sustained ocular drug delivery. The mechanistic study of drug release (Table S1) showed that the release followed the first-order kinetic equation (R^2^ > 0.95) and Langmuir equation (R^2^ > 0.95), indicating that drug release behavior is triggered by the dissolution of the gel matrix and a desorption process of physical adsorption.

### 2.3. In Vitro Cytotoxicity Assay

**Figure 5:**
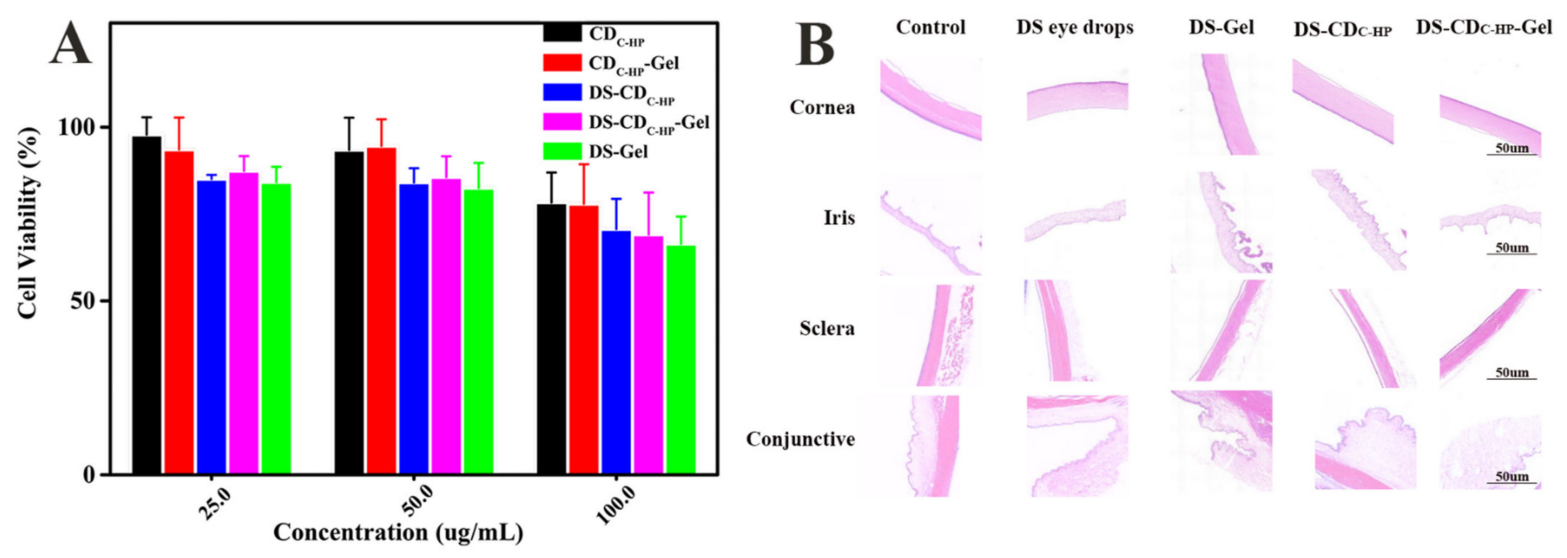
In vitro cytotoxicity of different formulations for 24 h (**A**); Histopathological microscopy of ocular tissues after treatment for a week (**B**).

Good cytocompatibility of nanocarriers is vital for drug delivery systems. Accordingly, MTT assays were used to evaluate the cytotoxicity of distinct formulations against HCE-T cells. The cell viability results are shown in Figure 5A, indicating no obvious cytotoxicity against HCE-T cells at any concentration in the present study. Even at high concentrations, the DS-CD_C-HP_-Gel did not show increased cytotoxicity compared with the simple DS-Gel group. Overall, DS-CD_C-HP_-Gel showed good cytocompatibility for use as a promising ocular drug delivery system.

### 2.4. Ocular Irritation Evaluation

For the eye, a special organ, irritation is a key factor for safety and patient compliance for ocular drug administration. After continuous administration of these formulations for 7 days, the rabbit eyes were observed and scored by the Draize scoring standard. Figure 5B shows that these tissues did not exhibit conjunctival hyperemia, corneal turbidity, or oedema inflammation and were not significantly different (*p* > 0.05, according to the Draize scoring standard) from the control group. The results suggested that DS-CD_C-HP_-Gel is promising for ocular drug delivery.

### 2.5. Ex Vivo Ocular Distribution Imaging

**Figure 6:**
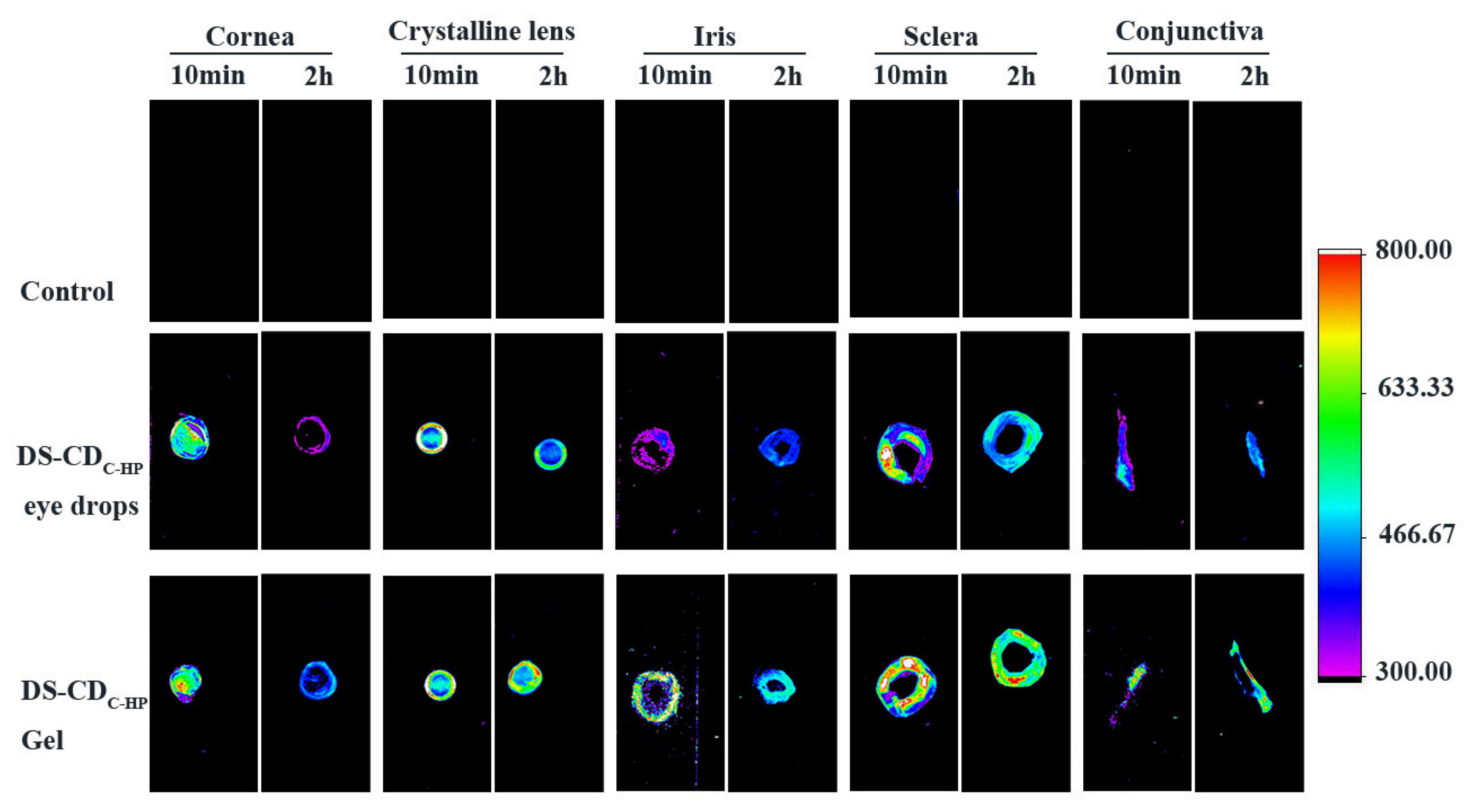
Fluorescence images of ex-vivo ocular tissues from DS-CD_C-HP_ eye drops and DS-CD_C-HP_-Gel treated rabbit eyes at 10 min and 2 h.

Based on the fluorescence properties of CD_C-HP_, the distribution of DS-CD_C-HP_ nanoparticles in ex vivo ocular tissues was investigated by in vivo fluorescence imaging technology. The ex vivo fluorescence distributions in ocular tissues of DS-CD_C-HP_ and DS-CD_C-HP_-Gel are shown in Figure 6. Notably, strong fluorescence signals of DS-CD_C-HP_ and DS-CD_C-HP_-Gel were observed in ocular tissues. As shown in Figure 6, the DS-CD_C-HP_-Gel emitted stronger fluorescence signals than the DS-CD_C-HP_ eye drops in the various tissues of the ocular at 10 min and 2 h. The DS-CD_C-HP_-Gel was equipped with a temperature-sensitive gel, which could prolong precorneal retention. This result was also observed in the fluorescence intensity of the cornea at 10 min and 2 h. In addition, we observed fluorescence signals at 2 h gathered in the crystalline lens, sclera, and conjunctiva, suggesting that CD_C-HP_ could enhance drug diffusion into the cornea. Furthermore, topical administration of CD_C-HP_ (DS-CD_C-HP_ eye drops and DS-CD_C-HP_-Gel) as a medication showed the strongest fluorescence intensity in the crystalline lens compared to the control group.

### 2.6. In Vivo Pharmacokinetics

#### 2.6.1. Elimination of DS in Tears

**Figure 7:**
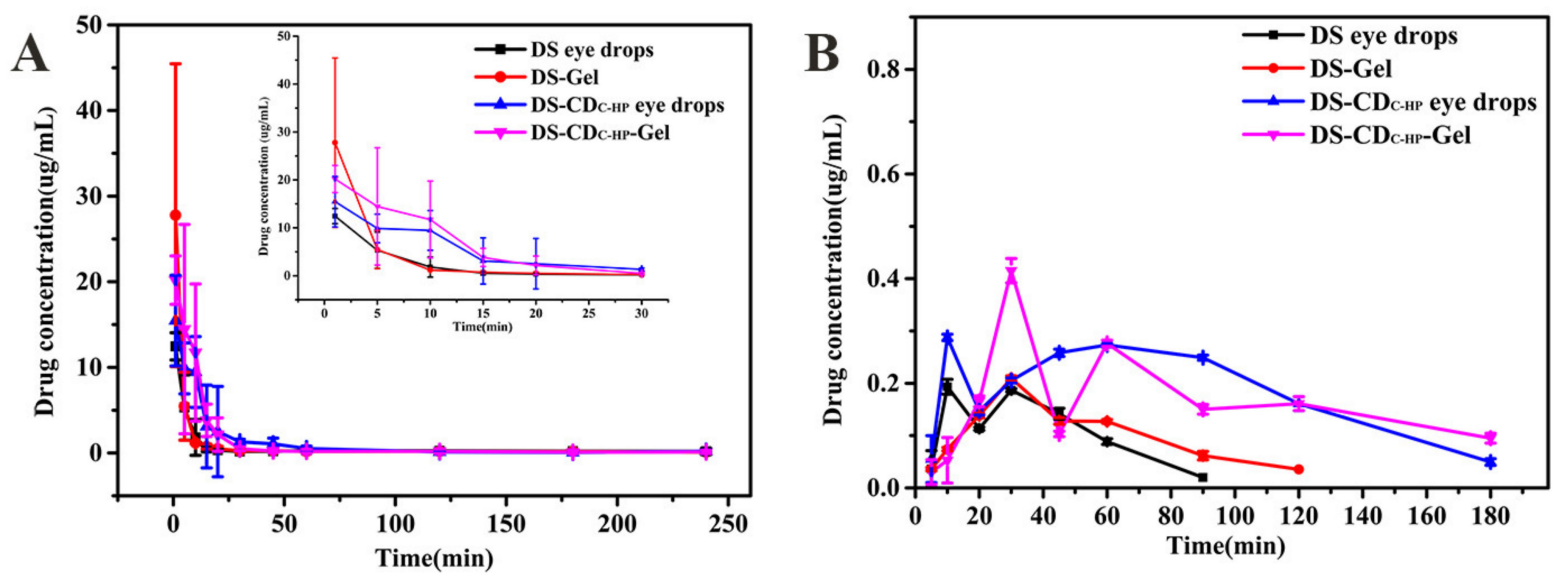
DS concentration vs. time profiles for the DS eye drops, DS-Gel, DS-CD_C-HP_ eye drops and DS-CD_C-HP_-Gel in lacrimal fluid (**A**) and the rabbit aqueous humor (**B**). Each value is the mean ± SD of three determinations.

The elimination of DS in tears of rabbits is shown in Figure 7A. As seen, the concentration of DS eye drops in tears only could be detected for 15 min due to rapid elimination. Although the concentration of DS-gel was higher than DS eye drops in tears for 1 min, it rapidly declined after 5 min. Compared with DS eye drops and DS-gel, the DS-CD_C-HP_-Gel showed a more sustained drug delivery with a high drug concentration within 30 min. This is mainly because the thermosensitive gel forms a hydrophilic gel layer on the surface of the eye, which prolongs the precorneal retention of DS. The elimination profiles of DS-CD_C-HP_ eye drops and DS-CD_C-HP_-Gel in tears corresponded to ex vivo fluorescence imaging studies.

#### 2.6.2. Absorption of DS in the Aqueous Humor

In Figure 7B, the change in the concentration of DS in the aqueous humor is displayed. The pharmacokinetic parameters of the DS eye drops, DS-Gel, DS-CD_C-HP_ and DS-CD_C-HP_-Gel that were obtained by DAS 2.0 software are shown in Table 2. A maximum concentration (C_max_) of 0.41 ± 0.02 μg/mL was measured in the rabbit aqueous humor after DS-CD_C-HP_-Gel administration and was 2.16-fold and 1.95-fold higher than those of DS eye drops and DS-Gel, respectively. Moreover, the area under the concentration-time curve (AUC_0-∞_, min·μg/mL) of DS-CD_C-HP_-Gel was significantly different from those of DS eye drops and DS-Gel (approximately 3.45-fold and 2.95-fold, respectively, *p* < 0.05). These results indicate that DS-CD_C-HP_-Gel has the potential to improve the bioavailability of ocular drug delivery.

## 3. Discussion

A composite ocular drug delivery system containing novel C-dots and a thermosensitive in situ gel was developed and characterized. The one-step hydrothermal method [17] was utilized to synthesize photoluminescent carbon dots (CD_C-HP_) consisting of biocompatible HA and CMC. HA is a dramatic carbon source and can be used as self-targeted imaging guided alone or accompanied by other passivating agents to prepare C-dots [24,27,37]. In the procedures, the use of two polymers (HA and CMC) with PEI to CD_C-HP_ by a one-step hydrothermal method has not yet been reported. The mechanism of the synthesis of CD_C-HP_ is the carboxyl groups of HA first polymerized with the amine of PEI or hydroxyl groups of CMC to form amide or ester polymers, and then, the polymers further carbonized and lost water to form CD_C-HP_ [38]. The optical properties, chemical structure, elemental composition, and surface state of CD_C-HP_ were investigated by UV-vis, fluorescence spectra, FTIR, XRD, XPS, TEM, and AFM. The results revealed that CD_C-HP_ was a graphene-like, amorphous, highly dispersed nanoparticle with a size of 0.2 nm that contained doped N atoms and emitted green fluorescence. Its high quantum yield (16.20%) may be related to the introduction of doped N atoms by CMC and PEI, which is consistent with the report by Gong et al [39]. Since photostability is a crucial characteristic in bioimaging, we further evaluated the 15-day emission behavior of CD_C-HP_ under sunlight. The photostability of CD_C-HP_ can be due to the existence of CMC and PEI polymers on the surface that prevent CD_C-HP_ particle aggregation. The fluorescence properties of CD_C-HP_ are mainly affected by the composition of atoms on its surface, leading to different energy of electron transition and different color fluorescence [15,27]. Unlike organic dyes, macromolecules with good biocompatibility were used as carbon sources, and the entanglement of macromolecules on the surface of CD_C-HP_ made the fluorescence relatively stable. In addition, the FTIR and XPS results suggested that the surface of CD_C-HP_ still retains the structural units of hyaluronic acid. HA is extremely water soluble and has a strong affinity for CD44 receptor overexpression on the surfaces of the cornea under inflammatory conditions [25], offering the possibility of further effective ocular drug delivery. Furthermore, cell receptor inhibition assay studies have shown self-targeted bioimaging (Figure S3), which is further proof of our hypothesis. Nontoxicity and bacteriostasis are important factors in the application of nanomaterials for ocular drug delivery systems [40]. The lack of cytotoxicity of CD_C-HP_ was attributed to the biocompatibility of the carbon source [15], and the ability to inhibit bacterial growth was attributed to the supercation (+38.33) of the CD_C-HP_ surface [19]. The results of in vitro cytotoxicity and antibacterial ability study showed that CD_C-HP_ had no cytotoxicity. This might be because the carbon source materials for the preparation of CD_C-HP_ were the biocompatible molecules CMC and HA. In addition, the large surface area to mass ratio of carbon dots greatly improves surface reactivity which contributes to good biocompatibility and antibacterial activity. Although a great deal of work has been used preclinical toxicological data of animal models to understand the toxicity of carbon dots to eyeball tissues [19,41], there is no sufficient evidence to demonstrate their safety in human eyes, because the biocompatibility and toxicity of carbon dots are closely related to their complex physicochemical properties and surface composition [42]. Therefore, it is still very important to develop an evaluation system that can fully evaluate the safety of this novel carbon dots for ocular drug delivery.

Furthermore, DS was selected as a drug model and was loaded onto CD_C-HP_ through electrostatic action to form a DS-CD_C-HP_ nanoparticle system. To further improve the DS duration and bioavailability from the DS-CD_C-HP_ nanoparticle system, DS-CD_C-HP_ was embedded in thermosensitive hydrogels to form a composite ocular drug delivery system [8]. The results of in vitro and in vivo studies revealed that DS-CD_C-HP_-Gel sustains release for 12 h with biphasic behavior and that DS-CD_C-HP_-Gel has no irritation to ocular tissue. This composite system improved bioavailability, possibly because the cationic DS-CD_C-HP_ particles and negatively charged corneal epithelial cells can form an electrostatic interaction and hydrogen bonds (-OH, -NH_2_ and -COOH) that reduce tear clearing [43]. In addition, DS-CD_C-HP_ particles combined with a thermosensitive gel prepared a platform for ocular drug delivery, which could prolong precorneal retention and reduce the contact angle (electrostatic interaction can be formed between positively charged nanoparticles and negatively charged corneal epithelial cells) [43], has the potential to improve the bioavailability of ocular drug delivery.

In this study we report a novel C-dots (CD_C-HP_) was synthesized by a simple one-step hydrothermal method of natural biomolecular materials (HA and CMC) and embedded it in thermosensitive hydrogels to form a composite ocular drug delivery system. On the basis of HA alone as carbon source, CMC and PEI were introduced to improve quantum yield and mass yield, and a new hybrid carbon dots with stable fluorescence characteristics, targeting and antibacterial activity was obtained. The functional carbon dots were combined with smart hydrogel to maximize the eye bioavailability of drugs.

## 4. Materials and Methods

### 4.1. Materials and Animals

Diclofenac sodium (DS) was acquired from the Wuhan Dongkangyuan Technology Co., Ltd. (Wuhan, Hubei, China). Polyethyleneimine (PEI, Mw = 10 kDa) was purchased from Macklin^®^ (Shanghai, China). Carboxymethyl chitosan (CMC, Mw = 197.17 kDa) was obtained from the Eisie Chemical Co., Ltd. (Jiaxing, Zhejiang, China). Hyaluronic acid (HA, MW < 10 kDa) was purchased from Freda (Jinan, Shandong, China). Poloxamer 407 (F407) and Poloxamer 188 (F68) were acquired from BASF (Ludwigshafen, Rhineland-palatinate, Germany). Hydroxypropyl methylcellulose E50 (HPMC E50) was a gift from the Anhui Shanhe Pharmaceutical Excipients Co., Ltd. (Huainan, Anhui, China). Other reagents involved in this work were all of analytical grade.

New Zealand albino rabbits (weighing 2.0–2.5 kg), having eyes without injury, were obtained from the Shenyang Pharmaceutical University Animal Center (Shenyang, Liaoning, China). All animal procedures in this study were conducted according to the principles of laboratory animal care and approved by the Animal Ethical Committee of Shenyang Pharmaceutical University.

### 4.2. Statistical Analysis

The data are shown as the mean ± SD, and analysis of variance with a significance of 0.05 indicated a statistically significant difference. The ocular pharmacokinetic parameters of DS in the aqueous humor and tears were evaluated by DAS 2.0 software (Shanghai, China).

### 4.3. Preparation of CD_C-HP_

CD_C-HP_ from hyaluronic acid and carboxymethyl chitosan was synthesized by a one-step hydrothermal method. Briefly, carboxymethyl chitosan with a degree of deacetylation ≥ 90% (200 mg) and hyaluronic acid sodium salt 6 kDa (200 mg) were dissolved in 20 mL of ultrapure water and stirred slowly until transparent. Then, 300 mg of PEI was added to the mixture and sufficiently mixed. This solution was transferred to a 50 mL Teflon-lined autoclave and heated at 170 °C for 12 h. After pyrolysis, the reaction products were cooled to room temperature and filtered through a 0.22 µm membrane filter to discard the pellet. The solutions were dialyzed against deionized water through a dialysis membrane (molecular weight cut off: 1000) for 12 h, with the water replaced every 4 h. The final products in the dialysis bag were a brown solution, which was then freeze-dried to obtain a brown solid. Finally, CDs were obtained and stored in the refrigerator for further use.

### 4.4. Physicochemical Characterization of the CD_C-HP_

FT-IR spectra were obtained using a Bruker IFS-55 spectrometer (Karlsruhe, Baden-Wurttemberg, Germany). The spectral properties of synthesized CD_C-HP_ were obtained using UV–vis absorption measurements of a 6100A instrument (Shanghai, China) from 200 to 700 nm. The fluorescence spectra were investigated by using a SpectraMax M3 microplate reader (San Jose, CA, USA). The crystallinity of CD_C-HP_ was studied by X-ray diffraction (XRD) on a Brooke D8 ADVANCE III 400 X-ray diffractometer (Karlsruhe, Baden-Wurttemberg, Germany) under a voltage of 35 kV and a current of 40 mA with a scanning angular scope of 3–50° (2θ) in a step width of 1°/min. The chemical compositions and chemical states of the products were tested by a Thermo 250Xi X-ray photoelectron spectroscopy (XPS) instrument (Waltham, MA, USA). The particle size and three-dimensional topography of the as-prepared CD_C-HP_ were evaluated using a Tecnai G2 F20 high-resolution TEM (HR-TEM) (Hillsboro, Oregon, USA) instrument and a Bruker Dimension Icon atomic force microscopy (AFM) instrument (Karlsruhe, Baden-Wurttemberg, Germany), respectively. Zeta potentials (ζ) of the CD_C-HP_ were measured using a Malvern Nano Zetasizer (Melvin, UK).

### 4.5. Preparation of Formulations

S eye drops as a negative control were prepared by dissolving 6 mg of DS in 10 mL of phosphate buffered saline (PBS) at a pH of 7.4. For DS-CD_C-HP_ eye drops, 1 mL of CD_C-HP_ solution (8 mg/mL) and 1 mL of DS solution (4 mg/mL) were mixed with PBS at a pH of 7.4 to form a 4 mL final solution system under stirring, followed by incubation for 12 h at room temperature. The resultant solution was filtered to remove impurities through a 0.22 µm microfiltration membrane and maintained in the dark for further use.

The formulation of the temperature-sensitive gel was determined by examining the gelation temperature (method: a precision thermometer with an accuracy of 0.1 °C was used to measure the temperature of the sample in the bottle. When the temperature was increased by 0.1 °C, the bottle was removed and quickly tilted to 45° to observe its flow). In this study, a gel matrix with a phase change temperature of approximately 34 °C was selected as the final formulation. DS-CD_C-HP_ was loaded into a temperature-sensitive hydrogel by using the swelling loading method [44,45]. Specifically, the mass fraction ratio of the dried temperature-sensitive hydrogel was 0.21:0.01:0.01 (poloxamer 407/poloxamer 188/HPMC E50; g/mL), which was dissolved in DS-CD_C-HP_ solution for 6 h at 4 °C and allowed to reach the equilibrium swelling state for further experiments. The gelling temperature and viscosity of the final formulation were studied after preparation.

The preparation process of DS-Gel was similar to that of DS-CD_C-HP_-Gel. In brief, 10 mL of PBS at a pH of 7.4 was employed to dissolve 6 mg of DS, and then, the temperature-sensitive hydrogel was swelled in by using a swelling-loading technique. The subsequent steps and conditions were the same as for the preparation of DS-CD_C-HP_-Gel.

### 4.6. Physicochemical Characterization of Formulations

#### 4.6.1. Characterization of Particles

The particle size (PS), polydispersity index (PDI), and zeta potential (ζ) were measured using a Malvern Zetasizer Nano, which determines the size distribution profile of small particles in suspension or in solution. The morphology was observed by AFM. A small amount of the diluted sample was dropped onto a mica plate, dried at room temperature and observed. FT-IR spectra were acquired using a Bruker IFS-55 spectrometer.

#### 4.6.2. Determination of Drug-Loading and Release

The DS loading efficiency (DLE) and drug loading content (DLC) were assessed by HPLC at a wavelength of 284 nm and the amount of DS was calculated according to a calibration curve. After preparing the DS-CD_C-HP_ eye drops, as described in Section 4.5, the solutions were dialyzed against 40 mL of deionized water through a dialysis membrane (molecular weight cut off: 1000) for 2 h. After this time, the solution inside the dialysis bag was DS-CD_C-HP_ complexes and the free DS was dialyzed into deionized water. The DLE and DLC of DS could be calculated according to Equations (1) and (2).

DLE (%) = (W_DS_ − W_free_)/W_DS_ × 100% (1)

DLC (%) = (W_DS_ − W_free_)/W_CD__C-HP_ × 100% (2)

W_DS_ denotes the total amount of DS in the loading experiment, W_free_ refers to the amount of DS dialyzed into the deionized water, and W_CD__C-HP_ represents the total quantity of CD_C-HP_.

The release profiles were assessed using a dialysis membrane diffusion technique in PBS at a pH of 7.4, respectively. In brief, accurately weighed amounts of samples containing 0.6 mg of DS were sealed in a dialysis bag (8000–14,000 Mw). Subsequently, the dialysis bag was immersed in 50 mL of PBS solution with a pH of 7.4 at 100 rpm and 34 ± 0.5 °C with continuous shaking. At specific time intervals, 1 mL of release medium outside the dialysis bag was removed, and the same volume of fresh PBS was replaced. The amount of DS released was quantified using HPLC at 284 nm.

### 4.7. In Vitro Cytotoxicity Assay

The in vitro cytotoxicity of DS eye drops, DS-Gel, DS-CD_C-HP_ eye drops and DS-CD_C-HP_-Gel against HCE-T cells was investigated using the MTT method. The detailed operation process was as follows: first, cells were incubated in 96-well plates for 24 h at a density of 1~2 × 10^4^ cells per well. Subsequently, the medium was discarded, and samples at different concentrations were replaced and incubated for 24 h. Then, 10 μL of MTT (5 mg/mL) was added to each well and incubated at 37 °C for 4 h. After discarding that culture medium, 200 μL DMSO was added to dissolve the formazan, and the absorbance of each well was determined at 490 nm by a SpectraMax M3 microplate reader (Molecular Devices, San Jose, CA, USA).

### 4.8. Ocular Irritation Evaluation

To evaluate potential ocular irritation of this composite ocular drug delivery system, the experiment was carried out three times a day for a week by the instillation (100 μL) of the formulations (DS eye drops, DS-Gel, DS-CD_C-HP_ eye drops and DS-CD_C-HP_-Gel) into the right eye, while the left eye received 100 μL of normal saline drops as a control group. After administration, the rabbits were euthanized by air injection intravenously into the marginal ear vein. Subsequently, the eyeballs were removed, washed with normal saline, fixed in formalin for 12 h, and embedded in paraffin. After staining with hematoxylin-eosin (H & E), the ocular tissues were observed under microscopy.

### 4.9. Ex Vivo Ocular Distribution Imaging

Based on the fluorescence properties of CD_C-HP_, the distribution of nanoparticles in eyeball tissues at different time points was investigated by in vivo fluorescence imaging technology (Olympus Corporation, Tokyo, Japan). In brief, two formulations (DS-CD_C-HP_ eye drops and DS-CD_C-HP_-Gel) and a blank control (normal saline, NS) were instilled into the corneas of rabbits. At specific time intervals (10 min and 2 h), rabbits were sacrificed by injection of air into ear margin veins. Afterwards, ocular tissues (including corneas, crystalline lenses, irises, scleras, and conjunctivas) were isolated carefully. During the experiment, ocular tissues were detected by using an in vivo imaging system (Carestream image station system FX Pro. Carestream Health. Toronto, Canada) equipped with light filter sets (E_x_/E_m_: 410/535 nm).

### 4.10. In Vivo Pharmacokinetics Study

#### 4.10.1. Elimination of DS in Tears

To demonstrate prolonged retention time of DS using the formulations (DS eye drops, DS-Gel, DS-CD_C-HP_ eye drops and DS-CD_C-HP_-Gel), the test of the elimination of DS in tears was studied. During the experiment, the tested rabbits were randomly divided into four groups (n = 3), and a single administration volume (100 μL, DS eye drops, DS-Gel, DS-CD_C-HP_ eye drops and DS-CD_C-HP_-Gel) was gently added into the conjunctival sac of the tested rabbit eye. After administration, the eyelids were gently kept closed 20 s manually. At regular time intervals, a filter paper strip (3 mm × 5 mm) was gently inserted into the lower eyelid of the eye for 10 s to collect tear fluids and weighed again. The samples were mixed with 200 mL of mobile phase in the tubes, vortexed for 90 s, and centrifuged for 15 min at 14,000 rpm. The concentration of DS in the supernatant was quantified by HPLC at 284 nm.

#### 4.10.2. Absorption of DS in the Aqueous Humor

To measure the amount of drug entering the eye, aqueous humor pharmacokinetic analysis was conducted using New Zealand white rabbits obtained from the Shenyang Pharmaceutical University Animal Centre. The tested rabbits were randomly divided into four groups (n = 3), and a single administration volume (100 μL, DS eye drops, DS-Gel, DS-CD_C-HP_ eye drops and DS-CD_C-HP_-Gel) was gently added into the conjunctival sac of the tested rabbit eye. After administration, eyelids were gently kept closed for 20 s manually. At regular time intervals (5, 10, 30, 45, 60, 90, 120, and 180 min), rabbits were sacrificed, and 100 mL of aqueous humor was collected using a 1 mL injection syringe. The samples were mixed with 200 mL of mobile phase (acetonitrile:deionized water:acetic acid = 70:30:0.3 (*v*/*v*/*v*)) in tubes, vortexed for 90 s, and centrifuged for 15 min at 14,000 rpm. The concentration of DS in the supernatant was quantified by HPLC at 284 nm.

## 5. Conclusions

Overall, a novel composite ocular drug delivery system in which drug C-dot particles are embedded in a thermosensitive in situ gel was developed and characterized. The one-step hydrothermal method was utilized to synthesize photoluminescent carbon dots (CD_C-HP_) consisting of biocompatible HA and CMC. Furthermore, diclofenac sodium was selected as a model drug, and negatively charged DS was loaded onto CD_C-HP_ through electrostatic action to form a DS-CD_C-HP_ nanoparticle system. The results indicated that this composite drug delivery system is safe and nonirritating for ocular applications. DS-CD_C-HP_-Gel not only provides bioimaging but also prolongs drug release, reduces tear elimination, and enhances aqueous humor bioavailability. This composite ophthalmic drug delivery system shows great potential in ocular drug delivery due to its ease of administration and better patient compliance, which results in reduced dosing frequency. More importantly, the successful development of this antimicrobial nanocarrier that can be loaded with anti-inflammatory drugs (NSAIDs) provides a new idea for ocular drug delivery systems.

## Figures and Tables

**Table 1:** Zeta potential (ζ), Particle size (PS) and particle size distribution index (PDI), loading efficiency (DLE) and loading content (DLC) of DS-CD_C-HP_. Values are mean ± SD (n = 3).

Sample	ζ (mV)	PS (nm)	PDI	DLE%	DLC%
DS	−17.17 ± 4.05	-	-	-	-
CD_C-HP_	+38.33 ± 2.47	-	-	-	-
DS-CD_C-HP_	+16.60 ± 0.43	90.12 ± 2.16	0.11 ± 0.02	84.37 ± 0.33	30.68 ± 0.10

**Table 2:** Pharmacokinetic parameters of DS in the aqueous humor of rabbits (n = 3).

Parameters	DS Eye Drops	DS-Gel	DS-CD_C-HP_ Eye Drops	DS-CD_C-HP_-Gel
T_max_ (min)	16.67 ± 11.55	30.00 ± 0.00	10.00 ± 0.00	30.00 ± 0.00
C_max_ (μg/mL)	0.19 ± 0.01	0.21 ± 0.00	0.28 ± 0.00	0.41 ± 0.02
t_1/2_ (min)	28.03 ± 2.97	34.46 ± 2.31	37.94 ± 1.36	78.20 ± 7.97
MRT_0−∞_ (min)	52.61 ± 3.15	64.88 ± 3.71	88.69 ± 1.48	137.49 ± 11.73
AUC_0−∞_ (min·μg/mL)	11.74 ± 1.17	13.72 ± 0.51	35.61 ± 0.97	40.54 ± 2.37

## Supplementary Materials

The following are available online at https://www.mdpi.com/article/10.3390/ijms22189934/s1.
