# Supplementary material for: A Novel Carbon Dots/Thermo-Sensitive In Situ Gel for a Composite Ocular Drug Delivery System: Characterization, Ex-Vivo Imaging, and In Vivo Evaluation
Source: Int J Mol Sci. 2021 Sep 14;22(18):9934. doi: 10.3390/ijms22189934 (PMC8464813; doi:10.3390/ijms22189934)
Supplement: Supplementary file 1 [file ijms-22-09934-s001.zip › ijms-1358490-supplementary.pdf]

Supplementary Material

A novel carbon dots /Thermo-sensitive in situ gel for a composite ocular drug delivery system: characterization, ex-vivo imaging, and in vivo evaluation

Lijie Wang[a]， Hao Pan[b]， Donghao Gu[a]， Haowei Sun[a]， Kai Chen[a], Guoxin Tan[a]， Weisan Pan[a,*]

a Shenyang Pharmaceutical University , Shenyang 110016, China
b Liaoning University , Shenyang 110036, China

Corresponding author: Professor Weisan Pan, Shenyang Pharmaceutical University, No.103, Wenhua Road, Shenyang 110016, China. E-mail addresses: pppwwwsss@163.com.

Table of contents:

2. *In vitro* cytotoxicity assay S1
3. Cell imaging S2
4. Storage stability S2
5. Mechanism study of drug release S2

Figure S1. MIC values of $CD_{C\text{-}HP}$ against *S. aureus, E. coli* and *P. aeruginosa*, (Mean±SD)........ S3

Figure S2. *In vitro* cytotoxicity of $CD_{C\text{-}HP}$ for 24 h and 48 h. S3

Figure S3. Fluorescence images of HCE-T cells incubated with $CD_{C\text{-}HP}$. S4

Figure S4. Changes in particle size and PDI of DS-$CD_{C\text{-}HP}$ in PBS at 4 °C for 14 days........ S4

Table S1. The kinetic model correlation coefficients (R^2) of the in vitro release curve........ S4

Experimental Section

1. Study on the ability to inhibit bacterial growth of the $CD_{C\text{-}HP}$

Three strains of *S. aureus*, *E. coli* and *P. aeruginosa* were obtained from the Institute of Food Science (Shenyang Pharmaceutical University, China) were selected as experimental strains. The ability to inhibit bacterial growth of the $CD_{C\text{-}HP}$ was tested by Disk-diffusion assay. First, a certain amount of the tested strains were inserted into the inclined medium of the test tube and incubated at 37 °C for 24 h to activate the tested strains. Then the bacterial suspension with the concentration of $10^7 \sim 10^8$ cfu·mL^{-1} was prepared with sterile saline for further use. Dipped the tested bacterial suspension to the inoculation rings and played on a nutrient agar plate. Filter paper was cut into circles of 5 mm diameters impregnated with different concentration samples of $CD_{C\text{-}HP}$ and placed on the above-mentioned nutrient agar plate. There were four pieces of circle paper on each plate (three parallels, blank control). Finally, in bacterial cultivation in 37 °C for 12 h, the whole experimental process with sterile distilled water as a blank control.

The MIC values of $CD_{C\text{-}HP}$ were determined by the serial microplate method. $CD_{C\text{-}HP}$ solution with a concentration of 12.8 mg/mL was serial diluted by Luria-Bertani (LB) medium prior to assay. The 96-well microplates were selected to assay the MIC of $CD_{C\text{-}HP}$. In brief, a series of concentration samples were obtained by two fold serial dilution of tested samples starting with a concentration of 12.8 mg/mL. 100 μL of bacteria suspension (*S. aureus, E. coli* and *P. aeruginosa*) was added to wells from column 1 to 11 (the column 12 was selected as blank) and then 100 μL of a series of concentration samples were added to the column 1 to 11. At the same time, control groups (LB, LB+ bacteria suspension, a series of concentration samples) were set up. Each concentration was triplicated (3 wells). The 96-well microplates were covered by preservative film to minimize the evaporation of the LB medium and incubated at 37 °C for 24 h. At the end of incubation, the absorbance values at 600 nm (OD_{600}) were determined by SpectraMax M3 (Molecular Devices, USA).

2. *In vitro* cytotoxicity assay

The *in vitro* cytotoxicity of $CD_{C\text{-}HP}$ against MCF-7, NIH-3T3, HCE-T and HRPEC

cells was investigated using the MTT method. The detailed operation process was as follows: firstly, cells were incubated in 96-well plates for 24 h with a density of 1~2×10^4 cells per well. Subsequently, the medium was discarded and samples at different concentrations were replaced and incubated for 24 h and 48 h. And then, 10 μL of MTT (5 mg/mL) was added to each well and incubated at 37 °C for 4 h. After discarding that culture medium, 200 μL DMSO was added to dissolve the formazan, and the absorbance of each well was determined at 490 nm by SpectraMax M3 microplate reader (Molecular Devices, USA).

3. Cell imaging

HCE-T (Zhejiang Meisen Cell Technology Co., Ltd, China) was selected as the model cell, and the bioimaging of CD_{C-HP} was observed by inverted fluorescence microscope (IX7I, Japan). The specific method was as follows: the cells were incubated in 24-well plates for 24 h with 10^5 cells per well and were divided into two groups. After that, one group was treated with free HA solution (MW < 10KDa, 10 mg/mL) incubation for 1 h to inhibit the CD44 receptor on the cell membrane surface. And then, the medium containing HA in the well was discarded and 20 μg/mL of CD_{C-HP} solution was added. At the same time, the other group added the same concentration of CD_{C-HP} solution. After incubation for 6 h, the medium was discarded and the cells were washed three times with PBS. Subsequently, cells were fixed with 4% paraformaldehyde at room temperature for 20 min, followed by stained with DAPI for 20 min to cell nuclei staining. Finally, the intracellular localization of samples observed with fluorescence microscope under the same conditions.

4. Storage stability

The DS-CD_{C-HP} were kept in dark place at 4 °C and the change of particle size distribution were measured at different time by Malvern Zeta sizer (UK).

5. Mechanism study of drug release

In order to further explain the drug release mechanism of DS-CD_{C-HP}-Gel, five kinetic models (zero-order, first-order, Higuchi, Freundlich and Langmuir kinetic models) were used to fit the release curves. The results are shown in Table S1. The fitting results showed that the release followed the first-order kinetic equation

(R^2 >0.95), indicating that drug release behavior is triggered by the dissolution of gel matrix. Furthermore, the fitting results of Freundlich and Langmuir equations showed that drug release followed Langmuir equation (R^2 > 0.95), indicating that drug release induced by a desorption process of physical adsorption. This further indicated that the binding mode of drug and carrier was electrostatic binding.

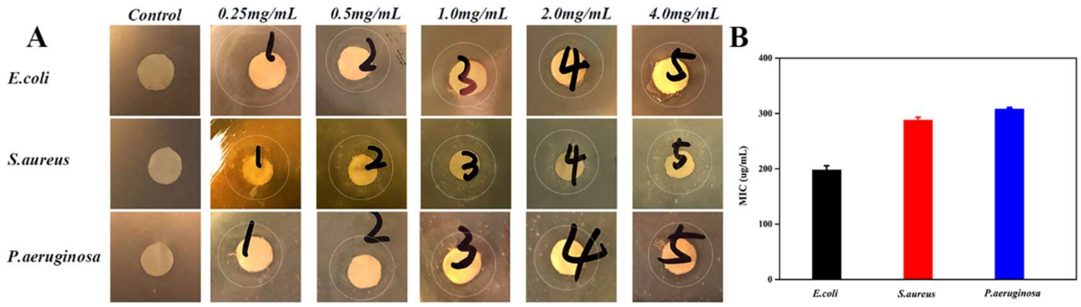

Figure S1. MIC values of $CD_{C\text{-}HP}$ against *S. aureus, E. coli* and *P. aeruginosa*, (Mean±SD).

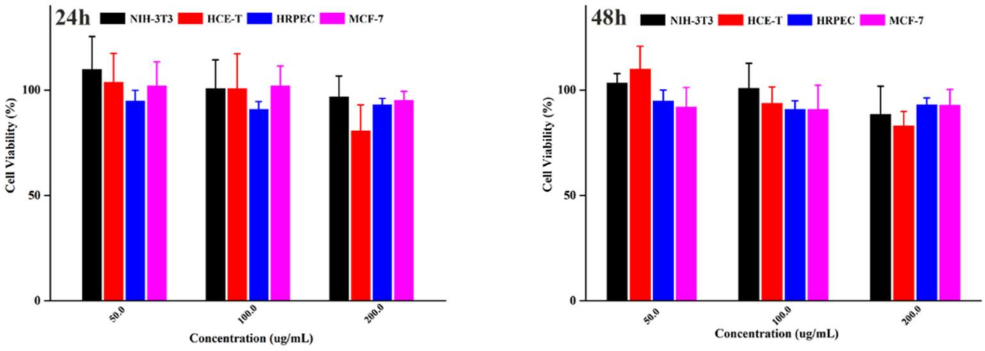

Figure S2. *In vitro* cytotoxicity of $CD_{C\text{-}HP}$ for 24 h and 48 h.

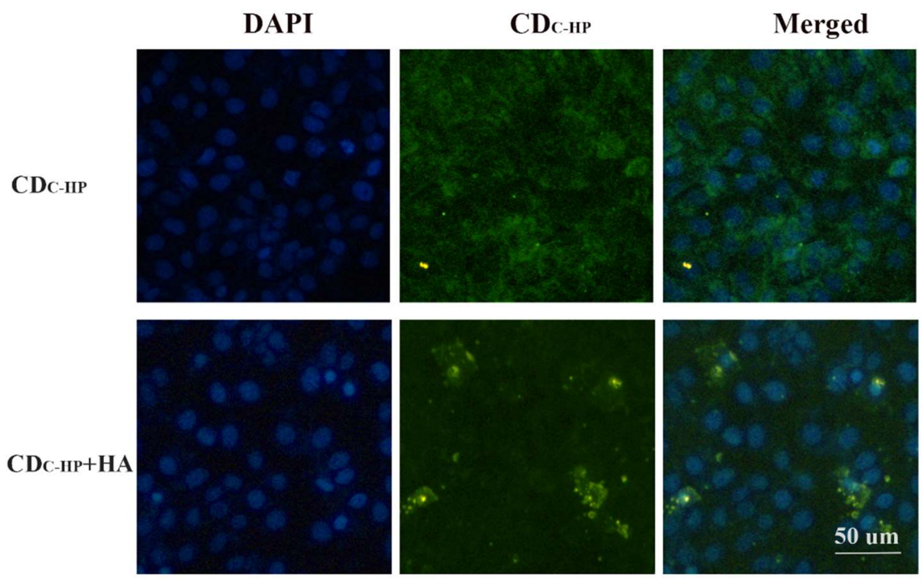

Figure S3. Fluorescence images of HCE-T cells incubated with CD_{C-HP}.

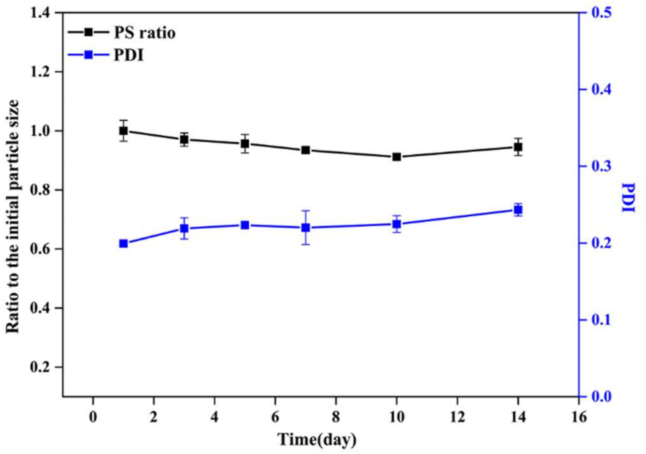

Figure S4. Changes in particle size and PDI of DS-CD_{C-HP} in PBS at 4 °C for 14 days**.**

Table S1. The kinetic model correlation coefficients (R^2) of the in vitro release curve.

Kinetic models	R^2
Zero-order	0.511
First-order	0.994
Higuchi	0.928
Freundlich	0.112
Langmuir	0.996
